# Prioritization of SNP markers for genomic prediction in closed beef cattle populations

**DOI:** 10.1093/tas/txaf166

**Published:** 2025-12-17

**Authors:** El Hamidi Hay

**Affiliations:** USDA Agricultural Research Service, Fort Keogh Livestock and Range Research Laboratory, Miles City, MT, 59301, United States

**Keywords:** genomic selection, high-density marker panels, SNP prioritization

## Abstract

With the advances in high-throughput technologies, genomic information is becoming readily available. This has led to whole genome sequences and denser single nucleotide polymorphism (SNP) panels being generated for more individuals. However, the increase in genomic information has shown little benefit in improving the prediction accuracy of genomic estimated breeding values (GEBV). One method to best utilize the increased amount of SNP information is to optimize the selection of informative SNP markers. In this study, genomic prediction of growth traits in two closed beef cattle populations using various prioritization techniques was evaluated. The first population used is Line 1 Hereford. The data consisted of 1192 animals with genotypes and phenotypes. The second population is a composite breed (50% Red Angus, 25% Charolais, 25% Tarentaise) and included of 2776 genotypes and phenotypes. The SNP prioritization methods adopted in this study were based on fixation index (Fst) and GWAS based SNP marker effects. Using a subset of prioritized SNP markers increased the accuracy for all three traits for the Line 1 Hereford population. On the other hand, using a weighted **G** matrix based on Fst and SNP effects did not increase the accuracy and in some instances decreased. Furthermore, the predication accuracy was higher in Line 1 Hereford which is an inbred population compared to the composite population. The study showed that prediction accuracy of GEBV can be improved with SNP prioritization, however it is population specific, trait specific and model specific. Moreover, this study highlights the importance of population structure in the prediction accuracy of GEBV.

## Teaser

This study explores how selecting the most informative DNA markers can boost the accuracy of predicting breeding traits in closed beef cattle populations, and why population structure plays a crucial role in genetic predictions.

## Introduction

Genomic selection has become a routine method in evaluating the genetic merit of livestock animals. This method utilizes dense single nucleotide polymorphism (SNP) panels to track genes and quantitative trait loci (QTL) under linkage disequilibrium (LD) (Meuwissen et al. 2001; VanRaden 2008; Harris and Johnson 2010). From a genetic improvement perspective, genomic selection resulted in a substantial increase in genetic gain across livestock populations. This improvement is due to the significant reduction in the generation interval and the increase in the accuracy of predicting genetic merit (VanRaden et al. 2009; Schefers and Weigel 2012).

With the recent advances in high throughput, SNP panels are becoming denser, and a larger number of individuals are being genotyped. Unfortunately, marginal improvement has been observed in the prediction accuracy of genetic merit with the increase of SNP panel density (Chang et al. 2019). Genomic selection is implemented either through multiple regression model or a mixed linear model. Increasing SNP density in the multiple regression method did not increase the prediction accuracy mainly due to the high dimensionality of the parameter space. For the mixed linear models, increasing the number of SNP to compute the genomic relationship matrix does not improve the estimation of additive relationships, therefore limiting the gain of prediction accuracy (Su et al. 2012). Due to these limitations, several methods were proposed to optimize the selection of informative SNP markers such as using SNP effects, fixation index (Fst) and biological relevance (Toghiani et al. 2017; Chang et al. 2019; Fragomeni et al. 2019; Toghiani et al. 2025). The results showed some promise in improving genomic prediction. However, most of these studies were simulation based, and more research is warranted to prioritize SNP markers using real data. Additionally, the improvement in accuracy is trait and population specific due to differences in genetic architecture. Therefore, the objective of this study is to evaluate the use of GWAS results and Fst values (markers under selection) as SNP prioritization tools and evaluate their effect on prediction accuracy for growth traits in two different beef cattle population structures.

## Materials and methods

### Data

This study used two closed beef cattle populations. The first population is Line 1 Hereford, a closed population that started in 1934 (MacNeil 2009; Leesburg et al. 2014). The pedigree consisted of 10,529 records with a subset of animals genotyped (1192). The current inbreeding level is 0.32.The second population is a composite breed referred to as Composite Gene Combination (CGC) composed of 50% Red Angus, 25% Charolais, and 25% Tarentaise (Newman et al. 1993). The population started in the late 1980s and remained a closed population. The pedigree consisted of 11,070 with 2776 animals genotyped. The inbreeding coefficient is 0.10.

Animals in Line 1 and CGC were genotyped with varying SNP density. All animals’ genotypes were imputed to a common SNP density panel using Beagle, Version 5.5 (Browning et al. 2021). The imputation accuracy was evaluated; across all SNP markers, the mean dosage *R*^2^ was 0.93 for CGC and 0.97 for Line 1.

Quality control was conducted by excluding SNP markers with minor allele frequency less than 0.05 and SNPs with Call Rate (CR_SNP_) < 0.90 and Fisher’s exact test *P*-value for Hardy–Weinberg Equilibrium (HWE) < 1 × 10^−5^. After quality control, the number of SNP genotypes consisted of 67,617 for Line 1 and 68,888 for CGC.

### Statistical analysis

The model used is GBLUP as follows:


y=Xb+Za+Wm+e,

where **y** is the vector of phenotypes, **b** is the vector of fixed effects consisting of sex and contemporary groups (birth year and dam age), **X** is the incidence matrix of fixed effects, **a** is the vector of additive genetic effects, **m** is the vector of maternal genetic effects. **Z** is the incidence matrix of random direct effects and **e** is the vector of residual effects. **W** is the incidence matrix of maternal effects. Additionally, it was assumed that additive genetic effects **a** and **m** were distributed as $N(0,\;G\sigma_{a}^{2})$ and $N(0,\;G\sigma_{m}^{2})$ respectively, with $\sigma_{a}^{2}$ being the direct genetic variance, $\sigma_{m}^{2}$ is the maternal genetic variance. The residuals **e** were distributed as $N(0,\;I\sigma_{e}^{2})$ with $\sigma_{e}^{2}$ being the residual variance. Due to the small size of the data, variance components were not estimated for the growth traits, but rather assigned values obtained from the literature (Nelsen et al. 1984; De Mattos et al. 2000; Koch et al. 2004). For Line 1, the following values were used for birth weight, $\sigma_{a}^{2}$ = 100.24 kg^2^, $\sigma_{m}^{2}$ = 95.18 kg, Cov(*a*, *m*) = −56.31 kg^2^ and $\sigma_{e}^{2}$ = 150.32 kg^2^. For weaning weight, the following values were used, $\;\sigma_{a}^{2}$ = 137.5 kg^2^, $\sigma_{m}^{2}$ = 93.4, Cov(*a*, *m*) = −48.0 kg^2^ and $\sigma_{e}^{2}$ = 302.1 kg^2^. For yearling weight, the following values were used, $\sigma_{a}^{2}$ = 563 kg^2^, $\sigma_{m}^{2}$ = 154, Cov(*a*, *m*) = −151 kg^2^ and $\sigma_{e}^{2}$ = 240 kg (Nelsen and Kress 1979).

For CGC, the following values were used for birth weight, $\sigma_{a}^{2}$ = 120.74 kg^2^, $\sigma_{m}^{2}$ = 37.63 kg^2^, Cov(*a*, *m*) = −27.00 kg^2^ and $\sigma_{e}^{2}$ = 250.7476 kg^2^. For weaning weight, the following values were used, $\sigma_{a}^{2}$ = 298 kg^2^, $\sigma_{m}^{2}$ = 168 kg^2^, Cov(*a*, *m*) = −165 kg^2^ and $\sigma_{e}^{2}$ = 258 kg^2^. For yearling weight, the following values were used, $\sigma_{a}^{2}$ = 563 kg^2^, $\sigma_{m}^{2}$ = 154, Cov(*a*, *m*) (direct × maternal covariance) = −151 kg^2^ and $\sigma_{e}^{2}$ = 240 kg (Bennett and Gregory 1996; Costa et al. 2011).

The analysis was carried out using BLUPF90+ (Version 1.1.0) program suite (Misztal 2002), preGSf90 to calculate the **G** matrix with varying structures (Aguilar et al. 2014) and postGSf90 (Aguilar et al. 2014) to carry out genome wide association study (GWAS). The genomic relationship matrix was calculated according to the method described in (VanRaden 2008) as the following:


G=MM' σs2 σa2=MIM'∑im2piqi

where **M** is the centered matrix of allele content, $p_{i}and\;q_{i}$ are the allele frequencies, ${\;\sigma }_{s}^{2}$ is the variance of SNP effects and **I** is the identity matrix with dimensions equal to the number of SNP.

### Fixation index-based SNP prioritization

In brief, Wright’s fixation index (Fst) is used to measure the level of genetic differentiation between populations based on change in allelic frequencies. The Fst values for Line 1 and CGC were calculated according to (Nei 1973; Chang et al. 2019) using PLINK software (Version 1.9) (Purcell et al. 2007). Each population was divided into two groups, where one group contained animals born before 2010 and the other group contained animals born after 2010. The birth year ranged from 1990 to 2023.

The top 10% Fst values of SNP were prioritized and either used alone in the genomic relationship matrix **G** or used with all SNP but assigned different weights based on their Fst values. The Fst weights were calculated according to Chang et al. (2019).

### GWAS-based SNP prioritization

The second prioritization method was based on GWAS results. After solving SNP effects from GEBV following Wang et al. (2012). The top 10% of SNP markers based on the absolute value of the effects were selected. These were included separately in the **G** matrix and included with all the SNP with varying weights. The SNP marker weighting was performed according to Wang et al. (2012) and (Bradford et al. 2017).

In brief, the weighting approach was the following:

A matrix of weights was included in the formula (VanRaden 2008) of the **G** matrix, to account for the heterogeneous SNP variance structure as the following:


$$\mathrm{var}(s)=\mathrm{diag}(D)=[\begin{array}{cccc} d_{1} & 0 & \ldots & 0\\ 0 & d_{2} & \ldots & 0\\ \ldots & \ldots & \ldots & \ldots \\ 0 & 0 & \ldots & d_{i} \end{array}]$$

Where var(**s**) is a diagonal matrix containing the variance of each individual SNP effect, **D** is a matrix of weights, and *d_i_* is the *i*^th^ diagonal element of **D** for the *i*^th^ SNP weight.

Therefore, a weighted relationship matrix can be written as:


$$G_{w}=\frac{\mathrm{MDM}}{\sum_{j=1}^{m}2p_{j}q_{j}},$$

where **M** is the centered matrix of allele content, $p_{i}and\;q_{i}$ are the allele frequencies.

The SNP effects were back solved from direct genomic values (DGV) and the weights were updated for 10 iterations based on preliminary results reported by (Wang et al. 2012).

### Genomic prediction accuracy

To test the prediction performance of the models, the dataset was split randomly into 2 sets containing 2/3 and 1/3 of the data for training and validation, respectively and the process was replicated 5 times.

The prediction accuracy was calculated as the correlation between predicted GEBV and corrected phenotype (**y**-**Xb**) in the validation dataset.

Other model performance criteria were evaluated; the slope of the regression (b) of GEBV on corrected phenotypes was calculated to test the degree of inflation or deflation of the genomic predictions. Also, the mean square error (MSE) was calculated to test the fit of the statistical models.

## Results and discussion

Summary statistics of the phenotypes are presented in Table 1. Line 1 population had lower average birth, weaning and yearling weights compared to CGC. This could be due to the high inbreeding level in the Line 1 Hereford population (Sumreddee et al. 2019, 2021) which has led to decreased growth.

**Table 1.** Summary statistics of the growth traits analyzed.

Trait	Line 1			CGC		
	*n*	Mean	SD	*n*	Mean	SD
**BW (kg)**	1192	33.19	4.73	2776	35.69	4.91
**WW (kg)**	1185	226.20	33.52	2735	236.98	34.37
**YW (kg)**	1163	331.85	66.87	2703	344.22	62.86

BW: birth weight; WW: weaning weight; YW: yearling weight.

**Fig. 1.**
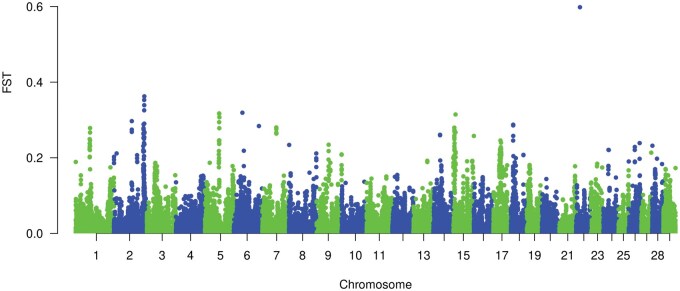
Manhattan plot of fst values for the line 1 population.

**Fig. 2.**
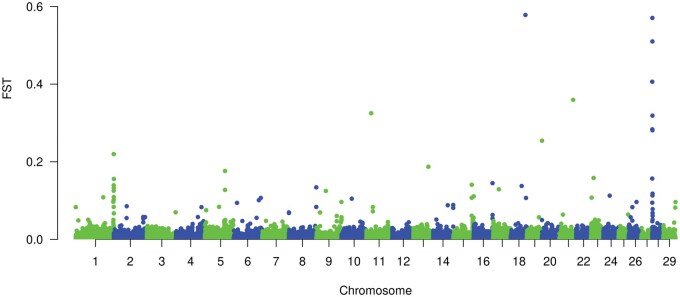
Manhattan plot of fst values for the CGC population.

The prioritization of SNP markers was carried out through two methods as described. The first method was Fst based. Manhattan plots of Fst values for both Line 1 and CGC are shown in Figs. 1 and 2 respectively. Several selection sweeps were detected. Surveying these regions under intense selection reveals various genes and QTL associated with fitness and production traits. For instance, in Line 1, a selection sweep located on 15.87 Mb of chromosome 22 harbored QTL associated with body size and growth in cattle along with genes such as *ANO10* which is involved in many physiological processes involved in skeletal muscle development (Chrysanthou et al. 2023). For CGC, a region under strong selection was detected on chromosome 19 in 63.43 Mb. This region contains genes such as *PRKCA* gene which is involved in growth and development in beef cattle (Chang et al. 2023).

**Fig. 3.**
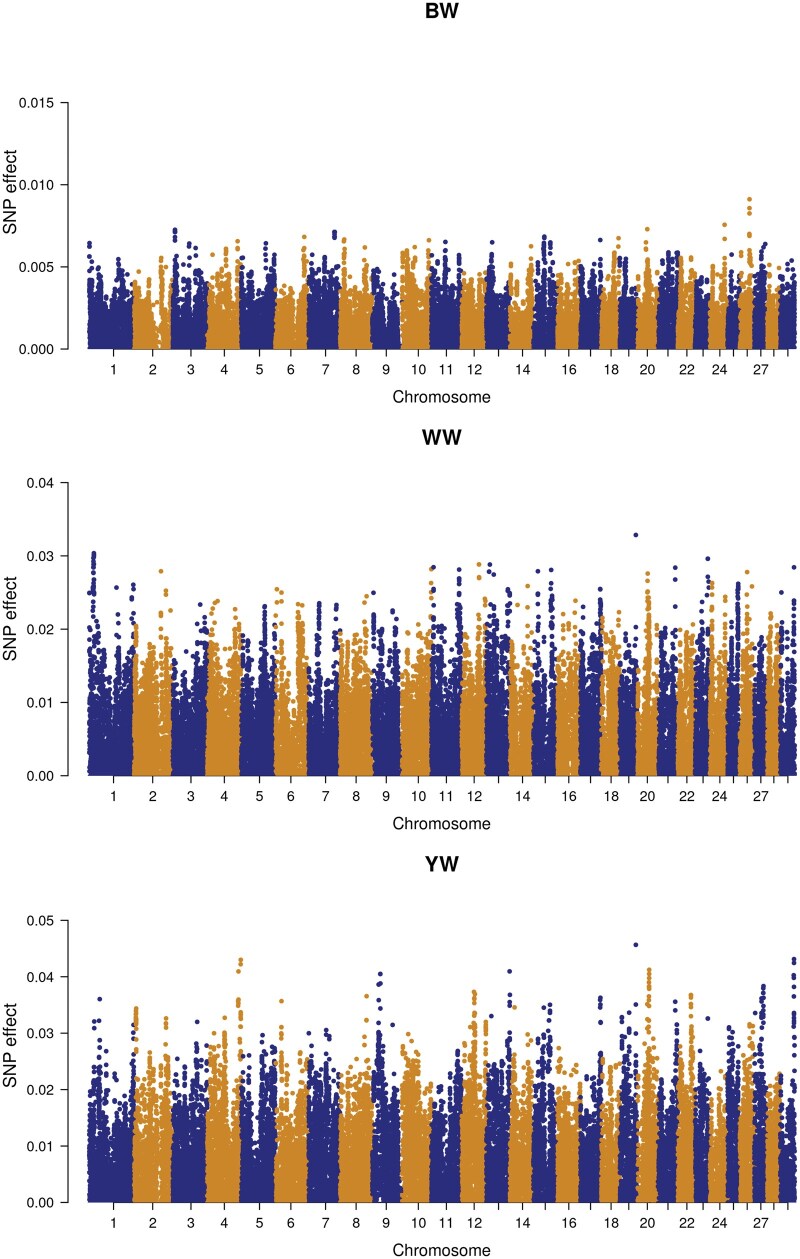
Manhattan plot of GWAS results for birth weight (BW), weaning weight (WW) and yearling weight (YW) in the line 1 population.

**Fig. 4.**
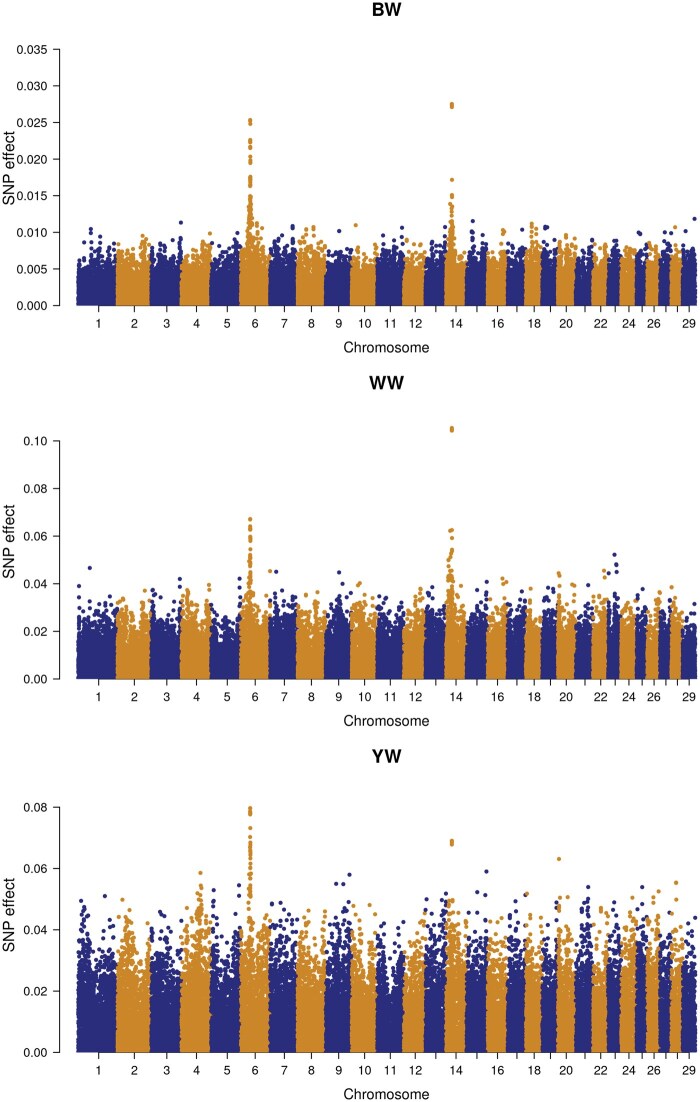
Manhattan plot of GWAS results for birth weight (BW), weaning weight (WW) and yearling weight (YW) in the CGC population.

The second prioritization method was based on GWAS results. Figures 3 and 4 show Manhattan plots of GWAS of all three growth traits. Here also, several associated SNP markers with strong effects on all three growth traits were detected and the top 10% regions included genes and QTL associated with growth traits.

Prioritizing or including SNP markers under selection or causative variants should increase the prediction accuracy of genomic estimated breeding values due to dimensionality reduction and genomic relationships reflecting QTL level similarity (Fragomeni et al. 2017; Chang et al. 2018; Chang et al. 2019; Fragomeni et al. 2019). Table 2 presents the prediction accuracies of all the three traits analyzed. For Line1, BW and YW prediction accuracies slightly increased when including only a subset of SNP markers through Fst and GWAS based prioritization. The accuracy for BW including all SNP markers was 0.43, and including only a subset of prioritized SNP markers the accuracy increased to 0.48. For WW, the accuracy remained unchanged. The prediction accuracy tends to remain unchanged once a sufficient number of SNP markers is used to capture relationships, since the main source of prediction accuracy in GBLUP comes from relationships in the **G** matrix. A simulation study by Chang et al. (2018) observed higher accuracies can be obtained with a reduced number of SNP markers. For CGC, prediction accuracy did not show an increase by including only prioritized SNP markers in the model except for a small increase seen in YW. This increase in prediction seen in Line 1 but not in CGC could be due to the differences in population structure. The Line 1 population is a highly inbred closed population with an effective population size of 8 (MacNeil 2009; Leesburg et al. 2014), therefore including a small number of informative SNP markers captures the genomic relationships among individuals. A study by Heidaritabar et al. (2016) showed that using a chicken population, including whole genome sequence (∼3.9 million of SNP) versus a 60k SNP panel did not increase the prediction accuracy. Another study in a pig population evaluating the inclusion of whole genome sequence data in prediction of GEBVs showed similar results (Ros-Freixedes et al. 2022). Population structure is an important factor in the accuracy of prediction of GEBV. A study by Pocrnic et al. (2016) showed the implications of effective population size and effective number of genome segments on the dimensionality of the genomic relationship matrix which in turn affect the accuracy of genomic predictions. In the inbred Line 1 population, the effective number of independent chromosome segments is lower. Therefore, the genomic relationship matrix has lower dimensionality (fewer large eigenvalues), so a smaller set of prioritized SNPs can capture most of the relationship structure among individuals. By contrast, in the composite population the effective population size is higher, haplotypes are shorter, relationships are more subtle, and **G** has higher dimensionality; hence more SNPs are required to reliably capture that structure and produce equivalent prediction accuracy. Accordingly, prioritizing SNP markers when computing **G** results in a greater proportional benefit in the inbred population than in the composite population (Pocrnic et al. 2016; Bradford et al. 2017; Abdollahi-Arpanahi et al. 2022).

**Table 2.** Accuracy of genomic prediction using prioritized SNP markers and weighted **G** matrix.

	Line 1				CGC			
Method^a^	nSNPs^b^	BW	WW	YW	nSNPs^b^	BW	WW	YW
**All SNP (Unweighted)**	67,617	0.43 (0.03)	0.38 (0.03)	0.32 (0.04)	68,888	0.44 (0.04)	0.35 (0.01)	0.30 (0.01)
**Pre-Selected (Fst)**	6761	0.48 (0.02)	0.38 (0.03)	0.35 (0.02)	6888	0.44 (0.03)	0.37 (0.02)	0.34 (0.01)
**Pre-Selected (SNP effect)**	6761	0.48 (0.01)	0.38 (0.02)	0.34 (0.03)	6888	0.45 (0.01)	0.36 (0.04)	0.30 (0.02)
**Weighted G (Fst)**	67,617	0.43 (0.02)	0.37 (0.01)	0.34 (0.03)	68,888	0.42 (0.02)	0.30 (0.02)	0.29 (0.03)
**Weighted G (SNP effect)**	67,617	0.45 (0.01)	0.36 (0.02)	0.32 (0.02)	68,888	0.40 (0.03)	0.32 (0.01)	0.30 (0.01)

a Prediction accuracy of the model is calculated as Pearson’s correlation between corrected phenotypes and genomic estimated breeding values in the validation data set.

b Number of SNP markers in each method. Standard deviations are in parentheses.

BW: birth weight; WW: weaning weight; YW: yearling weight.

For the prediction accuracies of a weighted genomic relationship matrix based on fixation index and GWAS, a decreasing trend in accuracy was observed. A small increase in accuracy was seen in Line 1 for BW (2 percentage points) using a weighted **G** matrix based on GWAS and for YW (2 percentage points) using a weighted Fst based **G** matrix. The results are not in concordance with the several simulation studies showing improved accuracy when weighing the **G** matrix was adopted (Fragomeni et al. 2017; Chang et al. 2019; Fragomeni et al. 2019). It is important to note that these studies have employed different methods of **G** matrix weighting. Furthermore, simulations often place QTL on the genotyped markers (or assume perfect linkage disequilibrium). In real cattle populations many QTL are poorly tagged by SNP chips (or have different LD across subpopulations). Weighting SNPs by FST or SNP effect estimates can therefore emphasize markers that are poor proxies of QTL in the validation set. This reduces the prediction accuracy compared with a uniform **G**.

Similarly, to including a selected subset of SNP makers, the weighting of the **G** matrix led to higher accuracy in the Line 1 population compared to the CGC composite population. A study by Lourenco et al. (2017) showed that weighted single-step GBLUP performed better in smaller populations (*Ne* = 20) than in larger populations (*Ne* = 100). The advantage of SNP weighing in smaller populations could be due to the limited amount of data required to accurately estimate SNP effects. The results of this study further highlight the important role of population structure in the prediction accuracy of genomic estimated breeding values.

Model fit and bias measurements are presented in Table 3. The three factors influencing the model fit and the slope of GEBV on the adjusted phenotypes are population structure, trait and the statistical model. When the **G** matrix was weighed by Fst or SNP effects, the regression slope was low compared to the other methods which is indicative of an underestimation of GEBV. Similar results were observed by Chang et al. (2019) when Fst was used to weigh the **G** matrix. This is contradictory to a study by Lopez et al. (2019) which has shown that SNP marker weighting of the **G** matrix reduces the deflation of GEBV. Weighting can induce changes in the scale and the signal in **G** so that it is no longer reflects the same additive relationships. Weighting SNPs with Fst tends to emphasize between-population differentiation (population structure) rather than within-family relationships. On the other hand, weighting SNPs by estimated effects can overfit or amplify noise (rare alleles/high sampling variance). Both effects can commonly produce biased (usually shrunken) GEBV (regression slope < 1). Chen et al. (2011), Eynard et al. (2015), Chang et al. (2019), Liu et al. (2020). Furthermore, randomly splitting the data can bias the results. Randomly splitting the data, full-siblings, half siblings and parent/offspring could end up in both validation and training sets causing the genetic relationships between two sets to be high and inflating accuracies. The weighting can therefore introduce bias to **G** to an already good **G** matrix which can cause the slopes to be less than 1.

**Table 3.** Mean square error and slope of the regression of genomic estimated breeding values on corrected phenotypes for different **G** matrix computation scenarios.

	Method	BW		WW		YW	
		β 1^a^	MSE^b^	β 1^a^	MSE^b^	β 1^a^	MSE^b^
**Line 1**	All SNP	0.95 (0.06)	1.31	1.09 (0.03)	4.62	0.92 (0.03)	19.34
	Pre-Selected (Fst)	0.91 (0.03)	1.44	1.12 (0.02)	4.71	0.91 (0.05)	20.26
	Pre-Selected (SNP effect)	0.92 (0.04)	1.22	0.92 (0.04)	4.40	0.90 (0.02)	22.84
	Weighted **G** (Fst)	0.78 (0.02)	1.41	0.67 (0.02)	5.01	0.69 (0.05)	23.29
	Weighted **G** (SNP effect)	0.82 (0.03)	1.30	0.72 (0.05)	5.20	0.66 (0.02)	25.37
**CGC**	All SNP	0.92 (0.02)	1.48	1.16 (0.01)	4.90	1.20 (0.02)	23.44
	Pre-Selected (Fst)	0.93 (0.02)	1.82	1.08 (0.05)	5.28	1.33 (0.03)	21.35
	Pre-Selected (SNP effect)	0.91 (0.04)	1.91	1.02 (0.04)	6.32	1.16 (0.01)	24.56
	Weighted **G** (Fst)	0.77 (0.02)	1.68	0.80 (0.01)	5.68	0.68 (0.02)	25.17
	Weighted **G** (SNP effect)	0.73 (0.01)	1.89	0.84 (0.02)	6.24	0.73 (0.01)	26.50

a Regression slope of genomic estimated breeding values on corrected phenotypes for animals in the validation dataset to measure the inflation of genomic prediction. Standard deviations are in parentheses.

b Mean squared error of genomic estimated breeding values and corrected phenotypes in the validation dataset as a measure of the fit of the model.

BW: birth weight; WW: weaning weight; YW: yearling weight.

Similarly to the regression slope, the mean square error showed that the weighting of the **G** matrix did not improve the fit of the model. By changing the scale or the direction of information in **G** such as Fst prioritization moves the estimated genomic breeding values further from the true breeding value. Further, even as the prediction accuracy increases, the MSE may not decrease since MSE penalizes bias heavily.

## Conclusions

Prioritizing SNP markers has the potential to improve genomic prediction accuracy either through a reduced number of markers or a weighted genomic relationship matrix. This allows extracting the full benefits of dense SNP panels or whole genome sequencing. Furthermore, this study highlights the effects of population structure on SNP marker prioritization in genomic prediction.

## Data Availability

The data supporting the findings of this study are available upon request from the author El Hamidi Hay: elhamidi.hay@usda.gov and with permission from the USDA Agricultural Research Service.

## List of Abbreviations:

SNP: single nucleotide polymorphism
QTL: quantitative trait loci
LD: linkage disequilibrium
Fst: fixation index
GBLUP: genomic best linear unbiased predictor
GEBV: genomic estimated breeding value
GWAS: genome wide association study
